# All-In-One: Advanced preparation of Human Parenchymal and Non-Parenchymal Liver Cells

**DOI:** 10.1371/journal.pone.0138655

**Published:** 2015-09-25

**Authors:** Melanie Werner, Sabrina Driftmann, Kathrin Kleinehr, Gernot M. Kaiser, Zotlan Mathé, Juergen-Walter Treckmann, Andreas Paul, Kathrin Skibbe, Joerg Timm, Ali Canbay, Guido Gerken, Joerg F. Schlaak, Ruth Broering

**Affiliations:** 1 Department of Gastroenterology and Hepatology, University Hospital of Essen, University Duisburg-Essen, Hufelandstr. 55, 45147, Essen, Germany; 2 Department of General-, Visceral-, and Transplantation Surgery, University Hospital of Essen, University of Duisburg-Essen, Hufelandstr. 55, 45147, Essen, Germany; 3 Department of Transplantation Surgery, Semmelweis University, Budapest, Hungary; 4 Institute of Virology, University Hospital of Essen, University of Duisburg-Essen, Virchowstr. 179, 45147, Essen, Germany; 5 Institute of Virology, University Hospital Düsseldorf, Düsseldorf, Germany; Vrije Universiteit Brussel, BELGIUM

## Abstract

**Background & Aims:**

Liver cells are key players in innate immunity. Thus, studying primary isolated liver cells is necessary for determining their role in liver physiology and pathophysiology. In particular, the quantity and quality of isolated cells are crucial to their function. Our aim was to isolate a large quantity of high-quality human parenchymal and non-parenchymal cells from a single liver specimen.

**Methods:**

Hepatocytes, Kupffer cells, liver sinusoidal endothelial cells, and stellate cells were isolated from liver tissues by collagenase perfusion in combination with low-speed centrifugation, density gradient centrifugation, and magnetic-activated cell sorting. The purity and functionality of cultured cell populations were controlled by determining their morphology, discriminative cell marker expression, and functional activity.

**Results:**

Cell preparation yielded the following cell counts per gram of liver tissue: 2.0±0.4×10^7^ hepatocytes, 1.8±0.5×10^6^ Kupffer cells, 4.3±1.9×10^5^ liver sinusoidal endothelial cells, and 3.2±0.5×10^5^ stellate cells. Hepatocytes were identified by albumin (95.5±1.7%) and exhibited time-dependent activity of cytochrome P450 enzymes. Kupffer cells expressed CD68 (94.5±1.2%) and exhibited phagocytic activity, as determined with 1μm latex beads. Endothelial cells were CD146^+^ (97.8±1.1%) and exhibited efficient uptake of acetylated low-density lipoprotein. Hepatic stellate cells were identified by the expression of α-smooth muscle actin (97.1±1.5%). These cells further exhibited retinol (vitamin A)-mediated autofluorescence.

**Conclusions:**

Our isolation procedure for primary parenchymal and non-parenchymal liver cells resulted in cell populations of high purity and quality, with retained physiological functionality *in vitro*. Thus, this system may provide a valuable tool for determining liver function and disease.

## Introduction

The liver plays an important role in metabolism and is involved in plasma protein synthesis, hormone production, detoxification, glycogen storage, decomposition of red blood cells, and other activities. It regulates biochemical reactions, including the synthesis and degradation of small and complex molecules that are essential for normal vital functions [1]. The liver mass is composed of 80% parenchymal cells (hepatocytes) and 20% non-parenchymal liver cells (NPC) [2]. Hepatocytes are metabolically active cells that execute such functions as glucose homeostasis, bilirubin excretion, protein synthesis, and secretion of major plasma proteins [3]. NPC are composed of Kupffer cells (KC), liver sinusoidal endothelial cells (LSEC), hepatic stellate cells (HSC), and rare cell types i.e. Pit cells [4, 5].

KC are the resident macrophage population within the lumen of sinusoids [6] and compose more than 80% of all tissue macrophages in the body [7, 8]. They control processes such as liver inflammation, hepatotoxicity, and injury [9]. Because of their localization and phagocytic activity, KC remove microorganisms, endotoxins, degenerated cells, immune complexes, and toxic agents from the blood [7, 10, 11]. LSEC line the sinusoidal wall and facilitate the exchange of fluids, solutes and particles between the liver sinusoidal blood and the space of Disse by dynamic filters, called fenestrae [12]. In addition, LSEC exhibit high endocytic capability and eliminate numerous substances from the blood by receptor-mediated endocytosis [13]. HSC reside in the subendothelial space [14] and were first described by Carl von Kupffer in 1876 as star-shaped cells that react to gold chloride [15]. In the healthy liver, stellate cells are the main cells responsible for the transport and storage of vitamin A in the body [14]. In diseased livers, HSC synthesize a large amount of extracellular matrix (ECM) proteins to stabilize the liver architecture, thereby promoting the process of liver fibrosis [16, 17]. Both hepatocytes and NPC seem to contribute to the induction of liver tolerance by their ability to sense pathogenic structures; they also contribute to immune regulation by functioning as antigen-presenting cells [18].

Isolating and culturing primary human hepatocytes (PHH) and NPC are essential for various purposes, such as the study of liver physiology and pathophysiology [19] and the study of hepatotoxicity [20]. Isolating human liver cells requires advanced experience with cell cultures and close cooperation with the surgical department; however, the quality of isolated cells depends on the condition of the donor tissue [21]. In 1972, Seglen *et al*. achieved an important advance by introducing the two-step perfusion technique for isolating rat liver cells [22]. Currently, diverse methods are available for isolating and cultivating single cell types (hepatocytes or NPC) from liver specimens [23–26].

When human liver cells are used, comparative studies profit from the use of cells isolated from liver tissues from the same patient. A recent study described the isolation of PHH and NPC from a single human liver specimen [27]. However, the purity of these various liver cells seems to be insufficient for detailed analyses and comparisons of cell type-specific and disease-related topics. Here, we describe an all-in-one preparation technique for obtaining functionally active PHH, KC, LSEC, and HSC of high purity and quality. This technique seems to be a beneficial tool for investigating liver function.

## Materials and Methods

### Liver tissues

The liver specimens (25-100g) were obtained from fresh tumor resections (n = 20). All patients provided written documentation of informed consent. The study conforms to the ethical guidelines of the 1975 Declaration of Helsinki and was approved by the Institutional Review Board (Ethics Committee) of the medical faculty at the University Duisburg-Essen.

### Isolation of PHH

**Fig 1:**
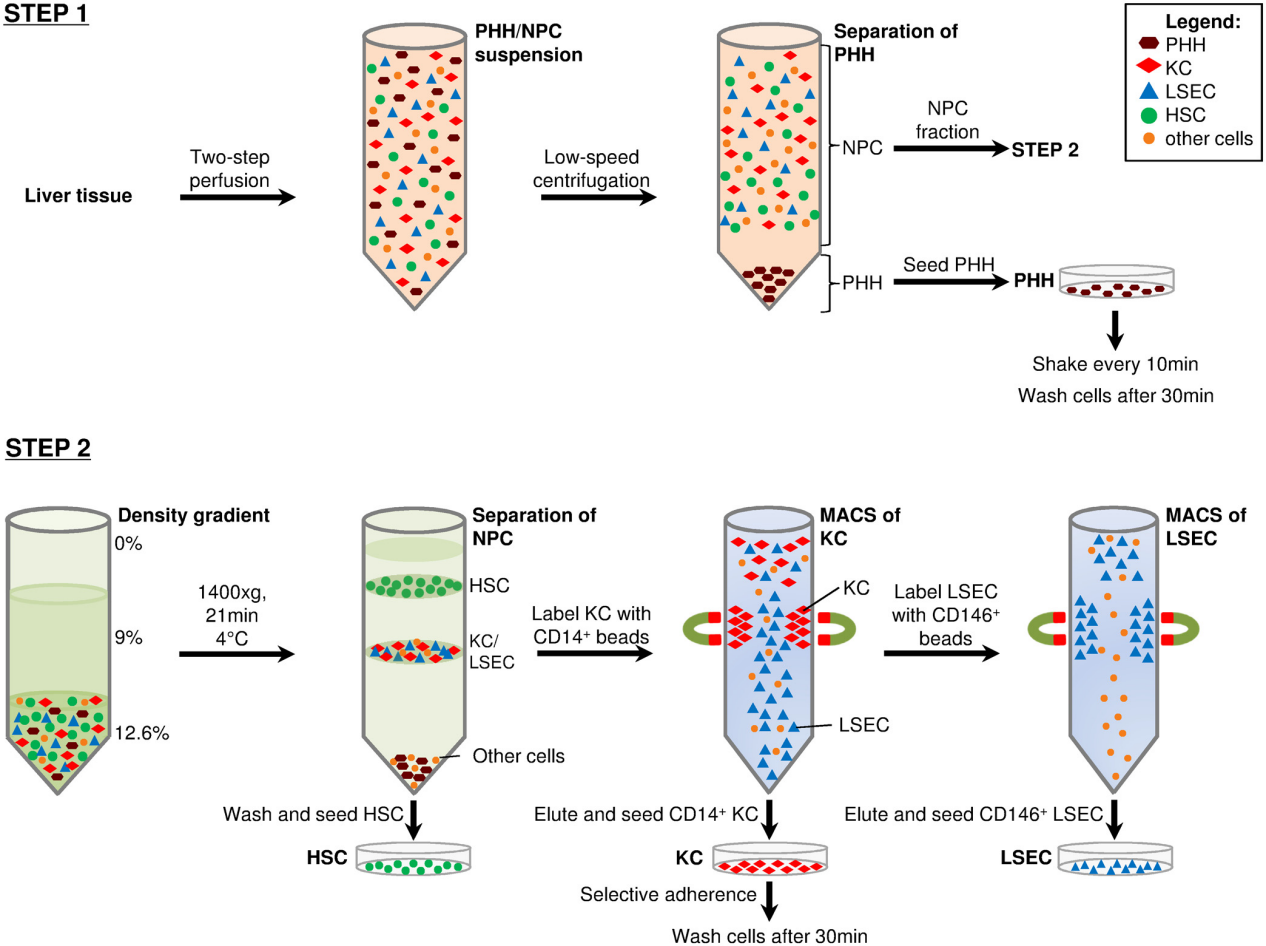
Preparation scheme for the isolation of primary liver cells. Liver cell suspensions were obtained by digesting liver tissues using collagenase two-step perfusion. PHH were pelleted by low-speed centrifugation at 30×g, 40×g and 50×g for 10min at RT. Supernatants containing NPC fraction were collected separately for later separation. PHH pellets were resuspended and seeded into dishes coated with collagen-I. Dishes were shaken every 10min and washed after 30-60min of incubation at 37°C and 5% CO_2_ atmosphere **(Step 1)**. NPC fraction was used to isolate and purify KC, LSEC, and HSC. The NPC suspension was pelleted and used for density gradient centrifugation (1400×g, 21min, 4°C) to separate KC and LSEC (lower layer) from the HSC (upper layer) fraction. HSC were seeded into a plastic culture flask. KC were purified by CD14^+^ MicroBeads followed by MACS. The flow through was collected for LSEC separation. CD14^+^ KC were eluted in culture medium and seeded into plastic culture plates. The medium was changed 30min after incubation (37°C and 5% CO_2_), to enhance the purity of KC by selective adherence. LSEC, which were present in the flow through, were labeled with CD146^+^ MicroBeads and MACS process was performed. Purified LSEC were seeded in collagen I-coated culture dishes **(Step2)**.

PHH and NPC were prepared from a single human liver specimen, as schematically illustrated in Fig 1. Liver cells were prepared under a biosafety hood according to a modified two-step perfusion technique described by Seglen [22]. In detail, the liver specimens were placed in a petri dish (14.5cm in diameter), and a cannula was positioned in an accessible hepatic vessel. The cannula was fixed, and remaining vessels were sealed with Histoacryl (Braun, Melsungen, Germany). The liver tissues were rinsed with perfusion solution (Ca^2+^- and Mg^2+^-free Hank’s balanced salt solution supplemented with 0.02mg/ml gentamycin and 20mM HEPES) pre-warmed to 37°C for 10 to 20min at a flow rate of 30 to 40ml/min. After nearly all blood had been flushed out, the livers were equilibrated with perfusion solution containing 0.5mM ethylene glycol tetraacetic acid (EGTA) for 10 to 20min in a recirculation approach. Collagenase type IV [0.6mg/ml] from *Clostridium histolyticum* (Sigma, Seelze, Germany) was dissolved in perfusion solution containing 5mM CaCl_2_ (Sigma), and the solution was sterilized through 0.45μm membrane filters (Pall Medical, Moeglingen, Germany). The duration of collagenase perfusion depended on tissue size and quality but did not exceed 20min. The obtained cell suspension was filtered through a 230μm-meshed cell strainer. PHH were then separated from NPC by low-speed centrifugation at gradually increasing rates (30×g, 40×g, and 50×g, for 10min). The cell pellets were resuspended in perfusion solution, whereas the supernatants were collected for the preparation of NPC, as described below. PHH were seeded into plates coated with collagen-I (BD Biosciences, Heidelberg, Germany) at a density of 1.25 to 2.5×10^5^ viable cells per cm^2^ by using Dulbecco’s modified Eagle’s medium (DMEM)/Ham’s F-12 (Biochrome, Berlin, Germany) supplemented with 10% fetal bovine serum (FBS; PAA, Pasching, Austria), 100U/ml penicillin (PAA), 0.1mg/ml streptomycin (PAA), and 2mM L-glutamine (Invitrogen, Darmstadt, Germany). Cells were incubated at 37°C under 5% CO_2_ atmosphere (standard conditions) and were manually shaken every 10min. The medium was changed to remove non-adhered cells 30 to 45min after seeding. The culture medium was replaced daily.

### Isolation of NPC

The NPC-containing cell suspension, collected during the PHH isolation process, was further used to isolate KC, LSEC, and HSC. Remaining PHH were removed from the NPC suspension by additional low-speed centrifugation (50×g, 2min, 4°C). The NPC-containing supernatants were collected. The cell suspension was pelleted by centrifugation (800×g, 10min, 4°C) and resuspended in Gey's balanced salt solution (GBSS) and iodixanol (OptiPrep, Axis-Shield, Oslo, Norway) to a final concentration of 12.6%. Afterwards, 5ml of the indicated suspension was placed in a 15ml polystyrene conical centrifuge tube (BD Biosciences) and overlaid with 5ml of a 9% iodixanol/GBSS solution followed by 2ml GBSS. After centrifugation at 1,400×g for 21min at 4°C with decreased acceleration and without breaks, the various cell-types were arranged according to their density. HSC were enriched in an upper cell layer, whereas KC and LSEC were separated as a second layer of higher density. Cell fractions were collected separately by pipetting. The KC/LSEC fraction was pelleted and KC were labeled with CD14^+^ MicroBeads (MiltenyiBiotec, Teterow, Germany) according to the manufacturer’s instructions. Cells were applied onto LS magnetic-activated cell sorting (MACS) columns (MiltenyiBiotec), which were placed within the magnetic field of a MACS separator and washed 3 times with MACS buffer (MiltenyiBiotec). CD14^+^ KC were eluted from the column by using 5ml DMEM supplemented with 10% FBS, 100U/ml penicillin, 0.1mg/ml streptomycin, and 2mM L-glutamine (KC culture medium). Viable KC were counted and seeded onto plastic culture plates at a density of 4 to 6×10^5^ cells per cm^2^ using indicated KC culture medium. Plates were gently washed 30min after seeding and were then incubated at 37°C and 5% CO_2_. The flow-through collected during KC separation was used to isolate LSEC. The LSEC were purified with a comparable MACS-based procedure using CD146^+^ MicroBeads. Cells were eluted in Endothelial Growth Medium 2 (PromoCell, Heidelberg, Germany) containing provided supplements, 100U/ml penicillin, and 0.1mg/ml streptomycin and were then seeded into culture dishes coated with collagen-I. After reaching 80% to 90% confluence, cells were detached by trypsin (PAA) and were propagated in culture plates coated with collagen-I. The HSC fraction, obtained by density gradient centrifugation, was seeded into an uncoated plastic culture flask with Stellate Cell Medium (ScienCell, Carlsbad, CA, USA) supplemented with supplied 10% FBS, 1% stellate cell growth supplement, 100U/ml penicillin, and 0.1mg/ml streptomycin. Once a confluence of 90% had been reached, cells were detached by trypsin and seeded onto culture plates using DMEM supplemented with 10% FBS, 100U/ml penicillin, 0.1mg/ml streptomycin, and 2mM L-glutamine.

### Identification of cell populations by immunofluorescence staining

Morphological characteristics were determined by phase contrast images acquired with an EVOS^TM^ XL Core Imaging System (AMG, Bothell, WA, USA). Cells were fixed with –20°C methanol/acetone (1:2) for 10min at 4°C and were washed with phosphate buffered saline (PBS). For intracellular staining, cells were permeabilized with 0.3% Triton X-100 in PBS for 30min, washed with PBS, and subsequently blocked with 2% bovine serum albumin in PBS for 1h. The cells were then incubated overnight at 4°C with a primary monoclonal mouse anti-albumin antibody (clone 188835, 1:1,000 dilution; R&D, Wiesbaden, Germany), a primary polyclonal rabbit anti-human CYGB antibody (1:500 dilution; Thermo Scientific, Bonn, Germany), a primary monoclonal anti-α-SMA antibody (clone 1A4, 1:700, Sigma), a directly coupled monoclonal mouse anti-human CD68-allophycocyanin (APC, clone Y1/82A; MiltenyiBiotec), a monoclonal mouse IgG2b-APC isotype control (clone IS6-11E5.11, 1:300 dilution; Sigma). After being washed with PBS, cells intended for staining with albumin were incubated for 3h at 4°C with a 1:1,000 dilution of secondary rat anti-mouse immunoglobulin (Ig) 2a (clone m2a-15F8) conjugated to phycoerythrin (PE; eBioscience, Frankfurt, Germany). Cells intended for staining with α-SMA and CYGB were incubated 3h at 4°C with a secondary polyclonal goat anti-mouse IgG coupled to DyLight594 (1:3000 dilution, Thermo Scientific) and a donkey anti-rabbit IgG coupled to DyLight488 (1:3000 dilution, Thermo Scientific). Negative control was accomplished by omitting the primary antibody. For the staining of extracellular markers, unspecific binding of antibodies was blocked with 1% FcR blocking reagent (MiltenyiBiotec) dissolved in PBS for 30min. Cells were incubated overnight at 4°C with the following directly fluorophore-conjugated monoclonal antibodies against discriminative cell markers or the respective isotype control (dilution of 1:200 in blocking solution): Mouse CD146-PE (clone 541-10B2; MiltenyiBiotec), mouse IgG_1_-PE (IS5-21F5; MiltenyiBiotec), mouse α-smooth muscle actin fluorescein isothiocyanate (SMA-FITC, clone 1A4; Sigma), or mouse IgG2a-FITC (clone UPC-10; Sigma). After incubation with antibodies for intracellular and extracellular staining, cells were washed with PBS and counterstained with 4′,6-diamidino-2-phenylindole (DAPI; Invitrogen). Slides were mounted in Fluoromount-G (eBioscience). Immunofluorescence staining was detected with a laser scanning microscope (LSM; Axiovert 100M; Zeiss, Jena, Germany) at 20× magnification. Image analysis was performed with LSM Image Browser (Zeiss).

### RNA isolation and one-step reverse transcription-quantitative real-time polymerase chain reaction

Total RNA was extracted from cultured cells with QIAzol Lysis Reagent (Qiagen, Hilden, Germany) and the RNeasy Mini Kit (Qiagen) according to the manufacturer’s protocols and RNA analysis was performed considering MIQE guidelines [28]. One-step reverse transcription-quantitative real-time polymerase chain reaction (RT-qPCR) was performed using QuantiFast SYBR Green PCR Kit (Qiagen). RNA quality was determined by Experion™ system (BioRad) and revealed an RNA quality indicator of 9–10. Amounts of 100-200ng RNA were used for RT-qPCR which reached a PCR efficiency of 88.3%. For gene expression of human *ACTB*, forward primer 5´-TCCCTGGAGAAGAGCTACGA-3´ and reverse primer 5´-AGCAATGTGTTGGCGTACAG-3´ were used. Expression levels of apolipoprotein B (*APOB*), asialoglycoprotein receptor 1 (*ASGR1*), *CYP3A4* (cytochrome P450, family 3, subfamily A, polypeptide 4), *CD163*, *CD14*, platelet/endothelial cell adhesion molecule 1 (*PECAM1*), von Willebrand factor (*VWF*), lymphatic vessel endothelial hyaluronan receptor 1 (*LYVE-1*), stabilin-1 (*STAB1)*, *STAB2* and *FCGR2B* (Fc fragment of IgG, low affinity IIb, receptor (CD32)), vinculin (*VCL*), desmin (*DES*), cytoglobin (*CYGB*), collagen type I alpha 1 (*COL1A1*) and lysyl oxidase-like 2 (*LOXL2*) were determined with commercially available primer sets (QuantiTec Primer Assay, Qiagen). Calculated copy numbers were normalized to *ACTB* and mean ± standard error of the mean (SEM) was determined. The expression of cell markers in the reference cell type was defined as 100%. The expression of these markers in the other cell types is expressed as fold change in %.

### Cytochrome P450 activity

PHH were seeded into 96-well plates and cultured for 1d. P450-Glo CYP3A4 Assay from Promega (Madison, WI, USA) was used for a non-lytic reaction according to the manufacturer’s protocol (n = 3) to assess CYP3A4 activity in PHH. Cells were stimulated with the CYP3A4 inducer dexamethasone [29] for 6-48h. Stimulation with the solvent ethanol (ETOH, 2% (v/v)) was performed as negative control. After incubation, PHH were exposed to 3μM Luciferin-IPA, here 2% (v/v) Dimethyl sulfoxide (DMSO) was added as an inhibitory control. Luminescence was detected after 1h of incubation by using the FLUOstar Omega from BMG LABTECH (Ortenberg, Germany). Mean relative luminescence signals were determined and net signals were calculated by subtracting the background luminescence signal (no cell control).

### Phagocytic activity

KC were incubated with 1μm amine-modified, yellow-green fluorescent (λ_ex_ = 470nm, λ_em_ = 540nm) latex beads (Sigma) at a dilution of 1:5,000 in DMEM supplemented with 10% FBS, 100U/ml penicillin, 0.1mg/ml streptomycin, and 2mM L-glutamine for 24h (n = 4). Cells were washed with PBS and fixed with –20°C methanol/acetone (1:2, 10min, 4°C). In addition, KC were stained for the marker CD68 as described above. Slides were mounted in Fluoromount-G. Images were captured with an LSM Axiovert 100M at 40× magnification. Images were analyzed and signal intensity was quantified using ImageJ software (http://imagej.nih.gov/ij).

### Uptake of acetylated low-density lipoprotein

LSEC were incubated with 5μg/ml acetylated low-density lipoprotein (AcLDL) conjugated to Alexa488 (Invitrogen) in culture medium for 1h (n = 4). Cells were washed with PBS and fixed with –20°C methanol/acetone (1:2, 10min, 4°C). Slides were mounted in Fluoromount-G. In addition, LSEC were stained for the surface marker CD146 as described above. An LSM Axiovert 100M was used at 40× magnification for imaging. Images were analyzed and signal intensity was quantified using ImageJ software.

### Detection of autofluorescence

HSC were seeded onto culture slides using DMEM supplemented with 10% FBS, 100U/ml penicillin, 0.1mg/ml streptomycin, and 2mM L-glutamine. After reaching 80% confluence, cells were washed with PBS and mounted with Fluoromount-G. Slides were directly used for detecting autofluorescent signals with a Leica TCS SP8 microscope with full-power ultraviolet (UV, 405nm) laser settings at 40× magnification (n = 4).

### Statistical analysis

Immunofluorescence staining of cell type—specific markers was performed in 5 independent experiments. For quantitative analysis, DAPI-stained, marker-positive, and marker-negative cells were counted in 10 independent images per cell type at 20× magnification. Cell numbers and purities are given as mean±SEM. The RNA expression of cell markers was determined in 5 independent experiments. These data are given in copy numbers as mean±SEM normalized to 100,000 copies of the reference gene *ACTB*. Differences between two groups were determined by Wilcoxon test, p<0.05 was considered to be statistically significant.

## Results

### Yields and purities of cultured primary liver cells

PHH and NPC composed of KC, LSEC, and HSC were isolated from fresh human liver resections. After liver cells had been isolated and purified, the yield of viable cells was determined (n = 10). The weight of liver tissues varied (25-100g) and led to cell yields of 2.0±0.4×10^7^ PHH, 1.8±0.5×10^6^ KC, 4.3±1.9×10^5^ LSEC, and 3.2±0.5×10^5^ HSC per gram liver tissue. After isolated cells had been plated, cell types were initially assessed for their unique morphology. Discriminative cell markers, which are specifically present in the respective cell type, could be determined at the protein level when examined by immunofluorescent staining (n = 5). For quantifying the purity of the various cell populations total cells (DAPI-stained), marker-positive cells, and marker-negative cells were counted in 10 independent images per cell type of 5 independent cell preparations (Table 1).

**Table 1:** Quantitative analysis of the purities of cultured cell populations.

Cell population	Cell marker	Number of marker-positive cells	Number of marker-negative cells	Purity
		(mean±SEM)	(mean±SEM)	(mean±SEM)
PHH (n = 5)	Albumin	529.4±192.2	21.2±6.0	95.5±1.7%
KC (n = 5)	CD68	299.0±106.4	17.8±5.3	94.5±1.2%
LSEC (n = 5)	CD146	489.6±86.3	10.2±5.0	97.8±1.1%
HSC (n = 5)	α-SMA	281.4±72.2	9.8±4.8	97.1±1.5%

Abbreviations: Primary human hepatocytes (PHH); Kupffer cells (KC); Liver sinusoidal endothelial cells (LSEC); Hepatic stellate cells (HSC); Cluster of differentiation (CD); α-smooth muscle actin (α-SMA); Standard error of mean (SEM).

### Characteristics of isolated PHH

**Fig 2:**
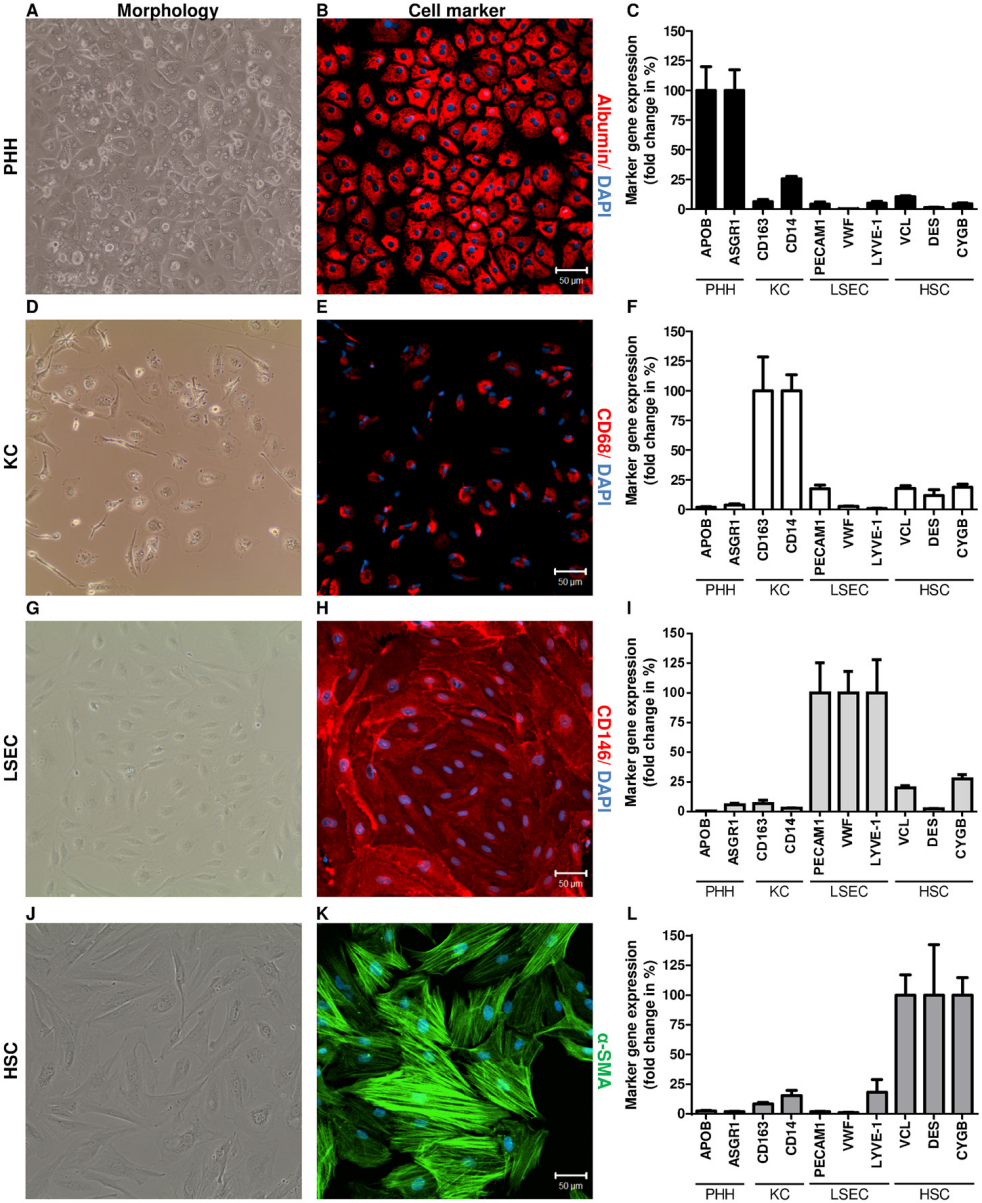
Identification of PHH and NPC. Cell morphology was examined by phase contrast microscopy using an EVOS^TM^ XL Core Imaging System (AMG). Isolated cell populations were identified by immunofluorescence staining of cell type—specific markers (n = 5). Nuclei were counterstained with DAPI (blue). Scale bar, 50μm. In addition, RNA was extracted from liver cells (n = 10) and gene expression of cell type-specific markers was determined by RT-qPCR. Copy numbers were normalized to the reference gene *ACTB*. The expression of cell markers in the reference cell type was defined as 100% (e.g. APOB in PHH). The expression of these markers in the other cell types is expressed as fold change in %. PHH exhibited a binucleated, polygonal shape (A) and strongly expressed albumin (B, red). In addition, PHH showed *APOB* and *ASGR1* gene expression (C). KC exhibited an irregular morphology (D) and expressed CD68 on protein level (E, red). Furthermore KC showed *CD163* and *CD14* gene expression (F). LSEC formed their unique morphology (G) and were identified by staining of CD146 (H, red). LSEC expressed the markers *PECAM1*, *VWF* and *LYVE-1* (I). HSC changed into myofibroblast-like cells (J) and were identified with anti-α-SMA antibody (K, green). Moreover, HSC were characterized by the gene expression of the markers *VCL*, *DES* and *CYGB* (L).

Cultured PHH, isolated from liver tissues using the two-step perfusion technique, exhibited the hepatocyte-related binucleated polygonal shape (Fig 2A), as assessed by phase contrast microscopy. Furthermore, PHH could be cultured for 10 days after isolation when the medium was supplemented with DMSO (2%) and epidermal growth factor (25ng/ml) upon day two of culture (S1 Fig). Hepatocytes were stained for the hepatocyte marker albumin. Imaging revealed a strong expression of albumin (Fig 2B) in 95.5±1.7% of cultured cells (Table 1). Furthermore, albumin staining was specific for hepatocytes and was not detectable in NPC populations (S2 Fig). In addition, RNA was extracted from cultured hepatocytes at day 2 after isolation and used for RT-qPCR. The hepatocyte-specific markers *APOB* (29,847.4±5,968.2) and *ASGR1* (1,687.8±292.7) [30–34] were expressed at a high mRNA level (Fig 2C), a finding underlining the identity of hepatocytes.

**Fig 3:**
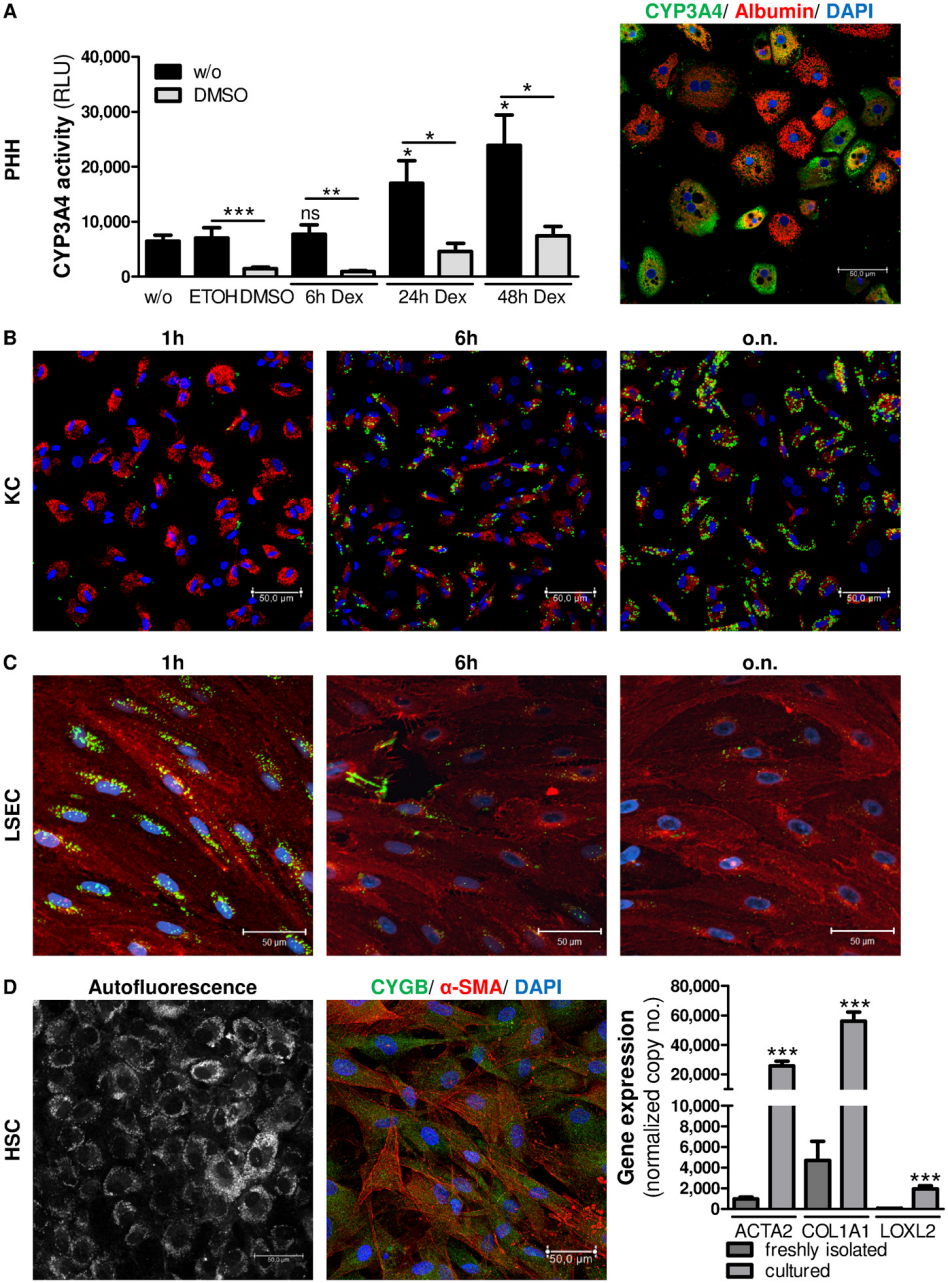
Functional activity of cultured cells. In PHH activity of CYP3A4 was determined after stimulation with 25μM dexamethasone for 6-48h. In addition CYP3A4 was inhibited by treatment with 2% DMSO. Cells were treated with ETOH or DMSO as negative controls. Relative light units (RLU) are given as mean±SEM (n = 3). Furthermore, CYP3A4 and albumin fluorescence imaging revealed a heterogeneous pattern of cultured PHH (A). KC exhibited strong phagocytic activity by the uptake of 1μm fluorescent latex beads (green) in a time-dependent manner (B). LSEC exhibited efficient endocytic capability, shown by the efficient incorporation of AcLDL (green) after 1h of incubation which was metabolized after incubation for 6h (C). HSC, cultured for a short time, were visualized by retinol (vitamin A) autofluorescence signals. Brightness reinforcement (BrightR) detection mode was used to amplify dim structures and make them accessible. To distinguish HSC from myofibroblasts, CYGB (green) and α-SMA (red) were fluorescently stained in the HSC population. Nuclei were stained with DAPI (blue). Images were taken at 40× magnification. Scale bar, 50μm. Furthermore, RNA was extracted from freshly isolated (uncultured) HSC and HSC cultured for 10 days (n = 6). Gene expression of *ACTA2*, *COL1A1* and *LOXL2* was determined by RT-qPCR. Data represent mean of copy numbers (mean±SEM) normalized to the reference gene *ACTB* (D). Asterisks indicate significant results (* p<0.05; ** p<0.01; *** p<0.001).

It is known that CYP3A4, which plays a crucial role in the oxidation of both xenobiotic and endogenous compounds, is expressed by hepatocytes [35]. Therefore, the physiological functionality of cultured hepatocytes was assessed by measuring the activity of the enzyme CYP3A4. To analyze the induction of CYP3A4 gene expression PHH were treated with 25μM dexamethasone for 6-48h and RNA was extracted (n = 3). CYP3A4 gene expression was assessed by RT-qPCR. CYP3A4 expression was significantly induced after stimulation with dexamethasone for 24h (p<0.022) and 48h (p = 0.011) compared to the control (S3 Fig). Moreover, dexamethasone-stimulated and unstimulated PHH were incubated with the CYP3A4 substrate and the CYP3A4 inhibitor DMSO for 1h, and enzyme activity was determined by measuring the generated luminescence (n = 3). CYP3A4 enzyme activity was induced in a time-dependent manner after 24h (p = 0.0358) and 48h (p = 0.0152) and was inhibited in the presence of DMSO (Fig 3A). Furthermore, CYP3A4 staining by immunocytochemistry (n = 3) revealed a heterogeneous distribution of CYP3A4 in PHH 24h after isolation (Fig 3A). In summary, the strong albumin production, together with the activity and inducibility of CYP3A4 in hepatocytes demonstrates the intact functionality of PHH in this early phase of culture.

### Characteristics of isolated human KC

Primary KC, prepared from liver cell suspensions by density gradient centrifugation and MACS, were cultured for 5–7 days until they formed their typical irregular morphology of both the elongated (stretched) and the oval shape and a bean-shaped nucleus [2, 6] (Fig 2D). Cultured KC were stained for the marker CD68. Fluorescence imaging revealed that 94.5±1.2% of the KC were CD68^+^ (Fig 2E, Table 1). Furthermore, CD68 protein expression was not detectable in PHH, LSEC and HSC populations (S2 Fig). In addition, after 5–7 days in culture total RNA was extracted from KC for determining the expression of known macrophage markers by RT-qPCR. The gene expression levels of *CD163* (3,502.6±1,003.0) and *CD14* (1,572.8±211.6) confirmed the identity of the cultured KC population (Fig 2F).

By their nature as macrophages, KC phagocytize particles of 200nm or more in diameter [25]. Therefore, KC were incubated with 1μm fluorophore-labeled, amine-modified polystyrene latex beads for 1h, 6h and 24h. Imaging data showed that incorporation of latex beads increased in KC over time (Fig 3B) and thus exhibited strong phagocytic activity *in vitro*. As a control LSEC were incubated with latex beads for 6-24h as well and were additionally stained for CD146. After imaging, the uptake of fluorophore-labeled latex beads was quantified in KC and LSEC in at least 5 images per cell type and incubation time. In comparison to LSEC, KC showed 5.8±0.4 (p<0.003) and 9.3±0.6 (p<0.002) fold higher latex bead-mediated fluorescence after 6h and 24h, respectively (S4 Fig).

### Characteristics of isolated human LSEC

The LSEC fractions, purified by labelling with CD146^+^ MicroBeads and MACS, were seeded into collagen-I-coated dishes and exhibited proliferative activity when resuspended in growth medium and seeded at sufficient density of >1×10^6^ cells per cm^2^. After reaching 80% to 90% confluence, approximately at day 4 after isolation, cells were detached by trypsin and were propagated in culture plates coated with collagen-I. The confluent LSEC monolayer was formed approximately at day 8 after isolation and cells exhibited the phenotypically endothelial-specific morphology (Fig 2G) [36, 37]. For identification of the cell population, cells were stained for CD146 and analyzed by fluorescence microscopy (n = 5) or flow cytometry (n = 3) (Fig 2H). CD146^+^ and CD146^−^ cells were counted in 10 independent images of 5 independent experiments. Direct immunofluorescence staining indicated a purity of 97.8±1.1% CD146^+^ LSEC (Table 1). Flow cytometry detected 93.5±2.6% CD146^+^ LSEC (S5 Fig). Furthermore, CD146 protein expression was not detectable in PHH, KC and HSC populations (S2 Fig). Moreover, total RNA was extracted at day 8 after isolation and LSEC identity was confirmed by the mRNA expression of known endothelial-specific markers [19]. Expression analysis showed that LSEC highly expressed *PECAM1* (18,656.3±4,766.7), *VWF* (6,073.2±1,093.4) and *LYVE-1* (1,430.0±400.6) (Fig 2I). However, gene expression of well-known LSEC markers like *STAB1*, *STAB2* and *FCGR2B* only reached marginal expression levels in the LSEC populations described here (data not shown). Therefore, we concluded that these markers were insufficient for a proper discrimination of primary isolated liver cell populations prepared with our method.

Because LSEC are known to preferentially eliminate soluble macromolecules of 200nm in diameter or lower by endocytosis [38], the functional activity of cultured LSEC was evaluated by the uptake of AcLDL *in vitro*. Therefore, LSEC were treated with 5μg/ml AcLDL for 1h, 6h and 24h. Afterwards cells were stained for CD146. Fluorescence imaging showed that cultured LSEC possessed a high ability to endocytose AcLDL after 1h in culture, a functional hallmark of LSEC. Importantly, the fluorescent signal of AcLDL decreased after 6h of incubation, indicating the metabolization of AcLDL by LSEC (Fig 3C). As a control KC were incubated with AcLDL for 1h to 6h as well and were additionally stained for CD68. After imaging, the uptake of fluorophore-labeled AcLDL was quantified in KC and LSEC in at least 5 images per cell type and incubation time. In comparison to KC, LSEC showed 11.1±2.9 (p<0.001) and 4.5±1.1 (p<0.001) fold higher AcLDL-mediated fluorescence after 1h and 6h, respectively (S6 Fig).

### Characteristics of isolated human HSC

The complete yield of HSC, separated from NPC suspension by density gradient centrifugation, was seeded into a 75cm^2^ plastic culture flask using stellate cell growth medium. Once 90% confluence was reached, cells were detached by trypsin and seeded into plastic dishes using DMEM supplemented with 10% FBS, 100U/ml penicillin, 0.1mg/ml streptomycin, and 2mM L-glutamine. Freshly isolated HSC exhibited a star-shaped morphology and transformed into an activated state, as identified by a myofibroblast-like phenotype within 10 days of cell culture (Fig 2J). HSC populations exhibited proliferative activity under usual humidified culture conditions (37°C, 5% CO_2_). It has been previously shown that HSC change into an activated state during primary cell culture [39]. To date, no consensus has been reached about markers suitable for quiescent and activated rodent and human HSC [40, 41]. In the current study α-SMA was used, a well-known marker of activated HSC. The analysis of fluorescence images showed that cultured HSC developed α-SMA cytoskeleton structures, a finding underlining the identity of activated HSC (Fig 2K). Quantitative analysis showed a purity of 97.1±1.5% for α-SMA—positive cells (Table 1). Furthermore, α-SMA protein expression was not detectable in PHH, KC and LSEC populations (S2 Fig). To validate the identity of HSC, gene expression of the HSC markers *VCL*, *DES* and *CYGB* was determined. Total RNA was extracted from cultured HSC approximately at day 10 after isolation and RT-qPCR was performed. HSC expressed high levels of *VCL* (18,660.9±3,158.9) and moderate levels of *DES* (363.7±155.0) and *CYGB* (333.8±48.8) (Fig 2L).

Because of their main characteristic of vitamin A storage, HSC can be identified by autofluorescence when excited with high-energy lasers [14, 42]. Because the activation of HSC is accompanied by the loss of vitamin A [43], HSC were used that had been cultured for only a few days. For the excitation settings, the UV laser was used at 405nm to detect autofluorescence signals of vitamin A. Moreover, the brightness reinforcement (BrightR) detection mode, an advantageous add-on of the Leica TCS SP8 microscope, was used to amplify dim structures and make them accessible. Imaging revealed various intensity levels of quickly fading autofluorescent retinoid droplets in the HSC population (Fig 3D). This phenomenon may indicate heterogeneity of isolated HSC population, as already been described [40, 44]. Furthermore cultured HSC were stained for CYGB and α-SMA to distinguish HSC from myofibroblasts as described elsewhere [45]. Imaging revealed high protein expression of CYGB in HSC. In addition, gene expression of functional cell markers was assessed in freshly isolated (uncultured) HSC and HSC cultured for 10 days (n = 6). The markers *ACTA2*, *COL1A1* and *LOXL2* were significantly induced after cultivation (Fig 3D).

## Discussion

For studying various aspects of liver function primary human liver cells, hepatocytes and NPC, must be of high quality. Diverse methods for isolating rodent and human liver cells have been described. However, most of these techniques focus on the preparation of a single cell type from liver tissues or biopsy specimens [23–26]. Here, we present a procedure for isolating primary hepatocytes, KC, LSEC, and HSC from a single human liver specimen, a procedure that results in cell populations with high purities and retained physiological functionality *in vitro*.

The choice of the digesting enzyme is important because the enzyme directly affects the viability, quality, and yield of isolated cells. Pronase and collagenase are frequently used to isolate HSC or KC [46]. For isolating a single cell type, pronase is used as a digesting enzyme because it provides selective destruction of hepatocytes [47] and thus directly enhances the cell quality of isolated NPC. However, Ikejima *et al*. [48] reported that pronase cleaves the CD14 receptor on KC, a receptor that is involved in the inflammatory response [49]. However, this phenomenon of cleaved CD14 was not observed after perfusion with collagenase. In our study, the human liver was dispersed by a two-step perfusion, based on the technique of Seglen, using Ca^2+^-free EGTA solution and Ca^2+^-containing collagenase IV solution [22]. In this procedure, the duration of collagenase perfusion is important and should not exceed 20 min. We and others have observed that over-digestion results in decreased cell viability and yield, whereas under-digestion prevents the separation of liver cells [50].

In the past, rodent and human NPC have been isolated with centrifugal elutriation methods [51] or with an iodixanol, Percoll, or Stractan density gradient [36, 47]. Because NPC are similar in density, the choice of the separation method is essential for high-quality cell preparations. Most recently, Pfeiffer *et al*. separated PHH from NPC fraction by low-speed centrifugation. NPC have been isolated as one fraction by a two-layer Percoll gradient (25% and 50%) centrifugation [27]. Afterwards, KC have been separated from LSEC and HSC by selective adherence to plastic. Furthermore, the authors have purified LSEC by CD31 MicroBeads and MACS. CD31^+^ LSEC have been seeded after elution from the MACS column. The CD31-depleted cell suspension contained HSC which have directly been seeded. Taken together, that approach resulted in similar cell yields, compared to the all-in-one cell preparation presented here. However, the limiting purities of KC (81.0±5.4%) and LSEC (81.0±1.7) populations underline the need of method improvements [27]. In contrast to the method used by Pfeiffer *et al*., we established a three-layer iodixanol density gradient to separate NPC. The advantage of this method is that KC and LSEC can be separated as one fraction from the HSC population. To date, there are limited surface markers suitable for separating HSC by MACS except for the stem/progenitor cell marker CD133, which can be used to isolate the CD133^+^ HSC subpopulation from rat and human liver tissue [52, 53]. A recently published method describes the isolation of an ultrapure HSC population by retinoic-based cell sorting of murine HSC [54]. To enhance the purity of the cells, commercially available magnetic beads specific for CD14 and CD146 were used to label KC and LSEC, respectively. The presented method provides cell yields comparable to those obtained by single and multiple cell type isolation methods. Cultured cells showed similar purities compared to single cell isolation techniques [55–57], whereas higher purities were obtained in comparison to methods describing simultaneous isolation of different liver cell types [27]. Here, purities of 95.5±1.7% PHH, 94.5±1.2% KC, 97.8±1.1% LSEC and 97.1±1.5% HSC could be reached, whereas the method described by Pfeiffer *et al*. revealed 92.3±3.2% PHH, 81.0±5.4% KC, 81.0±1.7% LSEC and 93.0±1.7% HSC determined by immunofluorescence staining of cell-specific antigens [27]. Furthermore, similar cell yields were observed in liver cell preparations from liver tissue with a different size of the starting material. This observation could be explained with variable perfusion efficiencies which depend on the availability of large vessels to place the cannula and a minimized cutting edge to allow an increase of pressure during perfusion. Therefore, tissue quality mainly depends on its previous location in the organ. In summary, it was demonstrated that PHH, cultured for short terms, and NPC retain their functional activity with an intact morphology *in vitro*, which means that these cells may be used in various experimental setups.

Our technique represents an adaptable tool with a wide range of additional perspectives. First, because stringent isolation of NPC is not necessary, hepatocytes alone can be prepared. CD133^+^ HSC have been isolated by labeling with MicroBeads and performing MACS after KC/LSEC has been removed by density gradient centrifugation [53]. Moreover, after PHH, KC, LSEC, and HSC have been separated; the CD14- and CD146-depleted cell suspension remaining at the end of the isolation procedure had been directly used or cryopreserved for the analysis of T cell or NK cell populations (unpublished data). Furthermore, isolated cells can be used for monoculture or co-culture experiments. In particular, Milosevic *et al*. and others used co-cultures of primary hepatocytes and KC, isolated from different rat livers, as a model to analyze xenobiotic metabolism and its pharmacological consequences [58]. When culture plate inserts with porous membranes (trans-well situation) are used, KC and hepatocytes can be kept spatially separated for investigating the initial role of KC and KC-derived soluble factors (exchanged via the medium) in hepatocyte toxicity. This approach is more similar to the *in vivo* situation than to direct cell-to-cell contacts.

Liver fibrosis is a consequence of chronic liver damage during the progression of chronic liver diseases. For instance, chronic viral hepatitis (hepatitis B and C), alcohol abuse, and nonalcoholic steatohepatitis (NASH) are the main causes of liver fibrosis and increase the risk of hepatocellular carcinoma [59]. Fibrosis is characterized by the continuous excessive production of ECM and collagen, which leads to permanent scarring and to organ malfunction that ultimately results in organ failure and death [60]. HSC have been identified as the main collagen-producing cell type in the liver [16]. Furthermore, KC and LSEC have been shown to have a substantial effect on the activation status of HSC [7, 61]. To date, the most effective therapy for hepatic fibrosis is the removal of the causative agent [62]. Additional studies are necessary to develop anti-fibrotic therapeutic agents for the robust prevention of fibrosis. Successful digestion of fibrotic liver tissue has already been described [63]. The all-in-one liver cell preparation presented here might also be used to investigate intercellular and molecular mechanisms of the fibrogenic process and therefore, might promote the development of effective therapeutic agents for patients with chronic liver damage.

In conclusion, we have developed an all-in-one technique for isolating primary human hepatocytes, KC, LSEC, and HSC from a single liver specimen. This technique results in cell populations of high purity with retained physiological activity *in vitro*. The preparation technique might be further adapted, thereby offering a wide range of additional perspectives. Isolated cells can be used as monocultures or co-cultures for investigations of the hepatotoxicity or pathophysiology of chronic liver diseases. The all-in-one liver cell preparation technique described here may provide a tool for investigating cellular and molecular mechanisms associated with liver function and disease *in vitro*.

## Supporting Information

S1 Fig Long-term culture of PHH. PHH were isolated from liver tissue and seeded into collagen-I-coated culture plates using DMEM/Ham’s F-12 supplemented with 10% FBS, 100U/ml penicillin, 0.1mg/ml streptomycin and 2mM L-glutamine. The medium was changed one day after seeding and then every second day. After two days of culture the medium was supplemented with DMSO (2%) and epidermal growth factor (25ng/ml). Cell morphology was exemplarily visualized by phase contrast microscopy using an EVOSTM XL Core Imaging System (AMG).
(TIF)

S2 Fig Cross-staining of cell type-specific markers in liver cell populations. Primary hepatocytes, KC, LSEC and HSC were isolated from human liver tissue (n = 3). Cultured cell populations were immunofluorescently cross-stained for cell type—specific markers (A). Negative controls were performed by omitting the primary antibody (albumin) or using isotype controls (B). Nuclei were counterstained with DAPI (blue). Images were captured at 20× magnification using laser scanning microscope (LSM; Axiovert 100M; Zeiss, Jena, Germany). Scale bar, 50μm.
(PDF)

S3 Fig Dexamethasone-induced CYP3A4 gene induction in PHH. Primary hepatocytes were isolated from human liver tissue (n = 3). One day post preparation PHH were stimulated with 25μM dexamethasone for 6-48h or ETOH for 48h (negative control). RNA was extracted and CYP3A4 gene expression was determined by RT-qPCR. Data represent mean of copy numbers (mean±SEM) normalized to the reference gene *ACTB*. Asterisks indicate significant results (* p<0.05; ** p<0.01; *** p<0.001).
(TIF)

S4 Fig Uptake of latex beads by KC and LSEC. KC (A) and LSEC (B) were incubated with fluorescently labeled latex beads (1μm in size, green) for 6-24h. After incubation cells were fixed and stained for CD68 (KC marker, red) or CD146 (LSEC marker, red), respectively. Nuclei were stained with DAPI (blue). Images were taken at 40× magnification. For quantification of the uptake efficiency intensities of latex bead fluorescence was measured in at least 5 images per cell population (C).
(TIF)

S5 Fig Identification of CD146-positive LSEC by FACS. LSEC were stained for CD146 expression and dead cells were excluded by the labelling with a viability dye. Flow cytometry was performed using the Navios flow cytometer (Beckman Coulter, Krefeld, Germany). Result analysis was performed using FlowJo (Treestar, Ashland, Oregon).
(TIF)

S6 Fig Uptake of AcLDL by KC and LSEC. KC (A) and LSEC (B) were incubated with fluorescently labeled AcLDL (green) for 1h and 6h. After incubation cells were fixed and stained for CD68 (KC marker, red) or CD146 (LSEC marker, red), respectively. Nuclei were stained with DAPI (blue). Images were taken at 40× magnification. For quantification of the uptake efficiency intensities of AcLDL fluorescence was measured in at least 5 images per cell population (C).
(TIF)

## Abbreviations

NPC: Non-parenchymal liver cells
KC: Kupffer cells
LSEC: Liver sinusoidal endothelial cells
HSC: Hepatic stellate cells
ECM: Extracellular matrix
PHH: Primary human hepatocytes
GBSS: Gey's balanced salt solution
MACS: Magnetic activated cell sorting
PBS: Phosphate buffered saline
α-SMA: α-smooth muscle actin
APOB: Apolipoprotein B
ASGR1: Asialoglycoprotein receptor 1
PECAM1: Platelet/endothelial cell adhesion molecule 1
VWF: Von Willebrand factor
LYVE-1: Lymphatic vessel endothelial hyaluronan receptor *1*
VCL: Vinculin
DES: Desmin
CYGB: Cytoglobin
COL1A1: Collagen type I alpha 1
LOXL2: Lysyl oxidase-like 2
SEM: Standard error of mean
AcLDL: Acetylated low-density lipoprotein

## References

[1] Jungermann K. Zonation of metabolism and gene expression in liver. Histochem Cell Biol 1995; 103:81–91. 7634156 10.1007/BF01454004

[2] Kmiec Z. Cooperation of liver cells in health and disease. Adv Anat Embryol Cell Biol 2001; 161:III–XIII, 1–151. 11729749 10.1007/978-3-642-56553-3

[3] McCuskey R. Anatomy of the liver In: Boyer TD, Manns MP, Sanyal AJ, editors. Zakim and Boyer’s Hepatology: a textbook of liver disease. Philadelphia, Pennsylvania, USA: Elsevier Sciences; 2012 p. 3–19.

[4] Blouin A, Bolender RP, Weibel ER. Distribution of organelles and membranes between hepatocytes and nonhepatocytes in the rat liver parenchyma. A stereological study. J Cell Biol 1977; 72:441–455. 833203 10.1083/jcb.72.2.441 PMC2110997

[5] Wisse E, van't Noordende JM, van der Meulen J, Daems WT. The pit cell: description of a new type of cell occurring in rat liver sinusoids and peripheral blood. Cell Tissue Res 1976; 173:423–435. 991252 10.1007/BF00224305

[6] Smedsrod B, De Bleser PJ, Braet F, Lovisetti P, Vanderkerken K, Wisse E et al Cell biology of liver endothelial and Kupffer cells. Gut 1994; 35:1509–1516. 7828963 10.1136/gut.35.11.1509 PMC1375602

[7] Kolios G, Valatas V, Kouroumalis E. Role of Kupffer cells in the pathogenesis of liver disease. World J Gastroenterol 2006; 12:7413–7420. 17167827 10.3748/wjg.v12.i46.7413 PMC4087584

[8] Bouwens L, Baekeland M, De Zanger R, Wisse E. Quantitation, tissue distribution and proliferation kinetics of Kupffer cells in normal rat liver. Hepatology 1986; 6:718–722. 3733004 10.1002/hep.1840060430

[9] Roberts R A, Ganey PE, Ju C, Kamendulis LM, Rusyn I, Klaunig JE. Role of the Kupffer cell in mediating hepatic toxicity and carcinogenesis. Toxicol Sci 2007; 96:2–15. 17122412 10.1093/toxsci/kfl173

[10] Clavien P A, Camargo CA Jr, Cameron R, Washington MK, Phillips MJ, Greig PD et al Kupffer cell erythrophagocytosis and graft-versus-host hemolysis in liver transplantation. Gastroenterology 1996; 110:1891–1896. 8964415 10.1053/gast.1996.v110.pm8964415

[11] Dini L, Pagliara P, Carla EC. Phagocytosis of apoptotic cells by liver: a morphological study. Microsc Res Tech 2002; 57:530–540. 12112436 10.1002/jemt.10107

[12] Braet F, Wisse E. Structural and functional aspects of liver sinusoidal endothelial cell fenestrae: a review. Comp Hepatol 2002; 1:1 12437787 10.1186/1476-5926-1-1 PMC131011

[13] Elvevold K H, Nedredal GI, Revhaug A, Smedsrod B. Scavenger properties of cultivated pig liver endothelial cells. Comp Hepatol 2004; 3:1–11. 15306034 10.1186/1476-5926-3-4 PMC514717

[14] Friedman S L. Hepatic stellate cells: protean, multifunctional, and enigmatic cells of the liver. Physiol Rev 2008; 88:125–172. 10.1152/physrev.00013.2007 18195085 PMC2888531

[15] Kupffer C v. Ueber Sternzellen der Leber. Archiv für Mikroskopische Anatomie 1876; 12:353–358.

[16] Moreira R K. Hepatic stellate cells and liver fibrosis. Arch Pathol Lab Med 2007; 131:1728–1734. 17979495 10.5858/2007-131-1728-HSCALF

[17] Tacke F, Weiskirchen R. Update on hepatic stellate cells: pathogenic role in liver fibrosis and novel isolation techniques. Expert Rev Gastroenterol Hepatol 2012; 6:67–80. 10.1586/egh.11.92 22149583

[18] Tiegs G, Lohse AW. Immune tolerance: what is unique about the liver. J Autoimmun 2010; 34:1–6. 10.1016/j.jaut.2009.08.008 19717280

[19] Lalor P F, Lai WK, Curbishley SM, Shetty S, Adams DH. Human hepatic sinusoidal endothelial cells can be distinguished by expression of phenotypic markers related to their specialised functions in vivo. World J Gastroenterol 2006; 12:5429–5439. 17006978 10.3748/wjg.v12.i34.5429 PMC4088223

[20] Liu Z X, Kaplowitz N. Role of innate immunity in acetaminophen-induced hepatotoxicity. Expert Opin Drug Metab Toxicol 2006; 2:493–503. 16859400 10.1517/17425255.2.4.493

[21] Godoy P, Hewitt NJ, Albrecht U, Andersen ME, Ansari N, Bhattacharya S et al Recent advances in 2D and 3D in vitro systems using primary hepatocytes, alternative hepatocyte sources and non-parenchymal liver cells and their use in investigating mechanisms of hepatotoxicity, cell signaling and ADME. Arch Toxicol 2013; 87:1315–1530. 10.1007/s00204-013-1078-5 23974980 PMC3753504

[22] Seglen P O. Preparation of isolated rat liver cells. Methods Cell Biol 1976; 13:29–83. 177845 10.1016/s0091-679x(08)61797-5

[23] Alabraba E B, Curbishley SM, Lai WK, Wigmore SJ, Adams DH, Afford SC. A new approach to isolation and culture of human Kupffer cells. J Immunol Methods 2007; 326:139–144. 17692868 10.1016/j.jim.2007.06.014

[24] Braet F, De Zanger R, Sasaoki T, Baekeland M, Janssens P, Smedsrod B et al Assessment of a method of isolation, purification, and cultivation of rat liver sinusoidal endothelial cells. Lab Invest 1994; 70:944–952. 8015298

[25] Bhogal R H, Hodson J, Bartlett DC, Weston CJ, Curbishley SM, Haughton E et al Isolation of primary human hepatocytes from normal and diseased liver tissue: a one hundred liver experience. PLoS One 2011; 6:e18222 10.1371/journal.pone.0018222 21479238 PMC3066224

[26] Chang W, Yang M, Song L, Shen K, Wang H, Gao X et al Isolation and culture of hepatic stellate cells from mouse liver. Acta Biochim Biophys Sin (Shanghai) 2014; 46:291–298. 24389643 10.1093/abbs/gmt143

[27] Pfeiffer E, Kegel V, Zeilinger K, Hengstler JG, Nussler AK, Seehofer D, et al Isolation, characterization, and cultivation of human hepatocytes and non-parenchymal liver cells. Exp Biol Med (Maywood). 2015;240: 645–656. 25394621 10.1177/1535370214558025 PMC4935273

[28] Bustin S A, Benes V, Garson JA, Hellemans J, Huggett J, Kubista M et al The MIQE guidelines: minimum information for publication of quantitative real-time PCR experiments. Clin Chem 2009; 55:611–622. 10.1373/clinchem.2008.112797 19246619

[29] McCune J S, Hawke RL, LeCluyse EL, Gillenwater HH, Hamilton G, Ritchie J et al In vivo and in vitro induction of human cytochrome P4503A4 by dexamethasone. Clin Pharmacol Ther 2000; 68:356–366. 11061575 10.1067/mcp.2000.110215

[30] Hubbard A L, Wilson G, Ashwell G, Stukenbrok H. An electron microscope autoradiographic study of the carbohydrate recognition systems in rat liver. I. Distribution of 125I-ligands among the liver cell types. J Cell Biol 1979; 83:47–64. 511941 10.1083/jcb.83.1.47 PMC2110443

[31] Hubbard A L, Stukenbrok H. An electron microscope autoradiographic study of the carbohydrate recognition systems in rat liver. II. Intracellular fates of the 125I-ligands. J Cell Biol 1979; 83:65–81. 511942 10.1083/jcb.83.1.65 PMC2110433

[32] Stockert R J. The asialoglycoprotein receptor: relationships between structure, function, and expression. Physiol Rev 1995; 75:591–609. 7624395 10.1152/physrev.1995.75.3.591

[33] Mason T M. The role of factors that regulate the synthesis and secretion of very-low-density lipoprotein by hepatocytes. Crit Rev Clin Lab Sci 1998; 35:461–487. 9885772 10.1080/10408369891234246

[34] Dixon J L, Ginsberg HN. Regulation of Hepatic Secretion of Apolipoprotein B-Containing Lipoproteins—Information obtained from Cultured Liver-Cells. J Lipid Res 1993; 34:167–179. 8381452

[35] Lacroix D, Sonnier M, Moncion A, Cheron G, Cresteil T. Expression of CYP3A in the human liver-evidence that the shift between CYP3A7 and CYP3A4 occurs immediately after birth. Eur J Biochem 1997; 247:625–634. 9266706 10.1111/j.1432-1033.1997.00625.x

[36] Daneker G W, Lund SA, Caughman SW, Swerlick RA, Fischer AH, Staley CA et al Culture and characterization of sinusoidal endothelial cells isolated from human liver. In Vitro Cell Dev Biol Anim 1998; 34:370–377. 9639099 10.1007/s11626-998-0018-9

[37] Elvevold K, Smedsrod B, Martinez I. The liver sinusoidal endothelial cell: a cell type of controversial and confusing identity. Am J Physiol Gastrointest Liver Physiol 2008; 294:G391–400. 18063708 10.1152/ajpgi.00167.2007

[38] Li R, Oteiza A, Sorensen KK, McCourt P, Olsen R, Smedsrod B et al Role of liver sinusoidal endothelial cells and stabilins in elimination of oxidized low-density lipoproteins. Am J Physiol Gastrointest Liver Physiol 2011; 300:G71–81. 10.1152/ajpgi.00215.2010 21030611 PMC3025507

[39] Sato M, Suzuki S, Senoo H. Hepatic stellate cells: unique characteristics in cell biology and phenotype. Cell Struct Funct 2003; 28:105–112. 12808230 10.1247/csf.28.105

[40] Cassiman D, Libbrecht L, Desmet V, Denef C, Roskams T. Hepatic stellate cell/myofibroblast subpopulations in fibrotic human and rat livers. J Hepatol 2002; 36:200–209. 11830331 10.1016/s0168-8278(01)00260-4

[41] Kawai S, Enzan H, Hayashi Y, Jin YL, Guo LM, Miyazaki E et al Vinculin: a novel marker for quiescent and activated hepatic stellate cells in human and rat livers. Virchows Arch 2003; 443:78–86. 12719976 10.1007/s00428-003-0804-4

[42] Wake K. "Sternzellen" in the liver: perisinusoidal cells with special reference to storage of vitamin A. Am J Anat 1971; 132:429–462. 4942297 10.1002/aja.1001320404

[43] Mezaki Y, Morii M, Hebiguchi T, Yoshikawa K, Yamaguchi N, Yoshino H et al The role of retinoic acid receptors in activated hepatic stellate cells. Med Hypotheses 2013; 81:222–224. 10.1016/j.mehy.2013.04.045 23688744

[44] D'Ambrosio DN, Walewski JL, Clugston RD, Berk PD, Rippe RA, Blaner WS. Distinct populations of hepatic stellate cells in the mouse liver have different capacities for retinoid and lipid storage. PLoS One. 2011;6: e24993 10.1371/journal.pone.0024993 21949825 PMC3174979

[45] Motoyama H, Komiya T, Thuy Le Thi Thanh, Tamori A, Enomoto M, Morikawa H et al Cytoglobin is expressed in hepatic stellate cells, but not in myofibroblasts, in normal and fibrotic human liver. Laboratory Investigation 2014; 94:192–207. 10.1038/labinvest.2013.135 24296877

[46] Knook DL, Seffelaar AM, de Leeuw AM. Fat-storing cells of the rat liver. Their isolation and purification. Exp Cell Res 1982; 139:468–471. 7084333 10.1016/0014-4827(82)90283-x

[47] Valatas V, Xidakis C, Roumpaki H, Kolios G, Kouroumalis EA. Isolation of rat Kupffer cells: a combined methodology for highly purified primary cultures. Cell Biol Int 2003; 27:67–73. 12713802 10.1016/s1065-6995(02)00249-4

[48] Ikejima K, Enomoto N, Seabra V, Ikejima A, Brenner DA, Thurman RG. Pronase destroys the lipopolysaccharide receptor CD14 on Kupffer cells. Am J Physiol 1999; 276:G591–8. 10070034 10.1152/ajpgi.1999.276.3.G591

[49] Heinzelmann M, Bosshart H. Heparin binds to lipopolysaccharide (LPS)-binding protein, facilitates the transfer of LPS to CD14, and enhances LPS-induced activation of peripheral blood monocytes. J Immunol 2005; 174:2280–2287. 15699163 10.4049/jimmunol.174.4.2280

[50] Zeng W Q, Zhang JQ, Li Y, Yang K, Chen YP, Liu ZJ. A new method to isolate and culture rat kupffer cells. PLoS One 2013; 8:e70832 10.1371/journal.pone.0070832 23967115 PMC3743898

[51] Irving M G, Roll FJ, Huang S, Bissell DM. Characterization and culture of sinusoidal endothelium from normal rat liver: lipoprotein uptake and collagen phenotype. Gastroenterology 1984; 87:1233–1247. 6092194

[52] Kordes C, Sawitza I, Muller-Marbach A, Ale-Agha N, Keitel V, Klonowski-Stumpe H et al CD133+ hepatic stellate cells are progenitor cells. Biochem Biophys Res Commun 2007; 352:410–417. 17118341 10.1016/j.bbrc.2006.11.029

[53] Beilfuss A, Sowa JP, Sydor S, Beste M, Bechmann LP, Schlattjan M et al Vitamin D counteracts fibrogenic TGF-beta signalling in human hepatic stellate cells both receptor-dependently and independently. Gut 2014. 10.1136/gutjnl-2014-307024 25134788

[54] Mederacke I, Dapito DH, Affo S, Uchinami H, Schwabe RF. High-yield and high-purity isolation of hepatic stellate cells from normal and fibrotic mouse livers. Nature Protocols 2015; 10:305–315. 10.1038/nprot.2015.017 25612230 PMC4681437

[55] Laba A, Ostrowska A, Patrzalek D, Paradowski L, Lange A. Characterization of human hepatocytes isolated from non-transplantable livers. Arch Immunol Ther Exp (Warsz). 2005;53: 442–453. 16314828

[56] Gerlach JC, Zeilinger K, Spatkowski G, Hentschel F, Schnoy N, Kolbeck S, et al Large-scale isolation of sinusoidal endothelial cells from pig and human liver. J Surg Res. 2001;100: 39–45. 11516203 10.1006/jsre.2001.6224

[57] Nakamura A, Ueno T, Yagi Y, Okuda K, Ogata T, Nakamura T, et al Human primary cultured hepatic stellate cells can be cryopreserved. Med Mol Morphol. 2010;43: 107–115. 10.1007/s00795-009-0484-5 20683699

[58] Milosevic N, Schawalder H, Maier P. Kupffer cell-mediated differential down-regulation of cytochrome P450 metabolism in rat hepatocytes. Eur J Pharmacol 1999; 368:75–87. 10096772 10.1016/s0014-2999(98)00988-1

[59] Bataller R, Sancho-Bru P, Gines P, Brenner DA. Liver fibrogenesis: a new role for the renin-angiotensin system. Antioxid Redox Signal 2005; 7:1346–1355. 16115040 10.1089/ars.2005.7.1346

[60] Wynn T A, Ramalingam TR. Mechanisms of fibrosis: therapeutic translation for fibrotic disease. Nat Med 2012; 18:1028–1040. 10.1038/nm.2807 22772564 PMC3405917

[61] DeLeve L D, Wang X, Guo Y. Sinusoidal endothelial cells prevent rat stellate cell activation and promote reversion to quiescence. Hepatology 2008; 48:920–930. 10.1002/hep.22351 18613151 PMC2695448

[62] Bataller R, Brenner DA. Liver fibrosis. J Clin Invest 2005; 115:209–218. 15690074 10.1172/JCI24282 PMC546435

[63] Broering R, Lutterbeck M, Trippler M, Kleinehr K, Poggenpohl L, Paul A, et al Long-term stimulation of Toll-like receptor 3 in primary human hepatocytes leads to sensitization for antiviral responses induced by poly I:C treatment. J Viral Hepat. 2014; 21: 480–490. 10.1111/jvh.12174 24750363

