# Supplementary figures and images for: All-In-One: Advanced preparation of Human Parenchymal and Non-Parenchymal Liver Cells
Source: PLoS One. 2015 Sep 25;10(9):e0138655. doi: 10.1371/journal.pone.0138655 (PMC4583235; doi:10.1371/journal.pone.0138655)

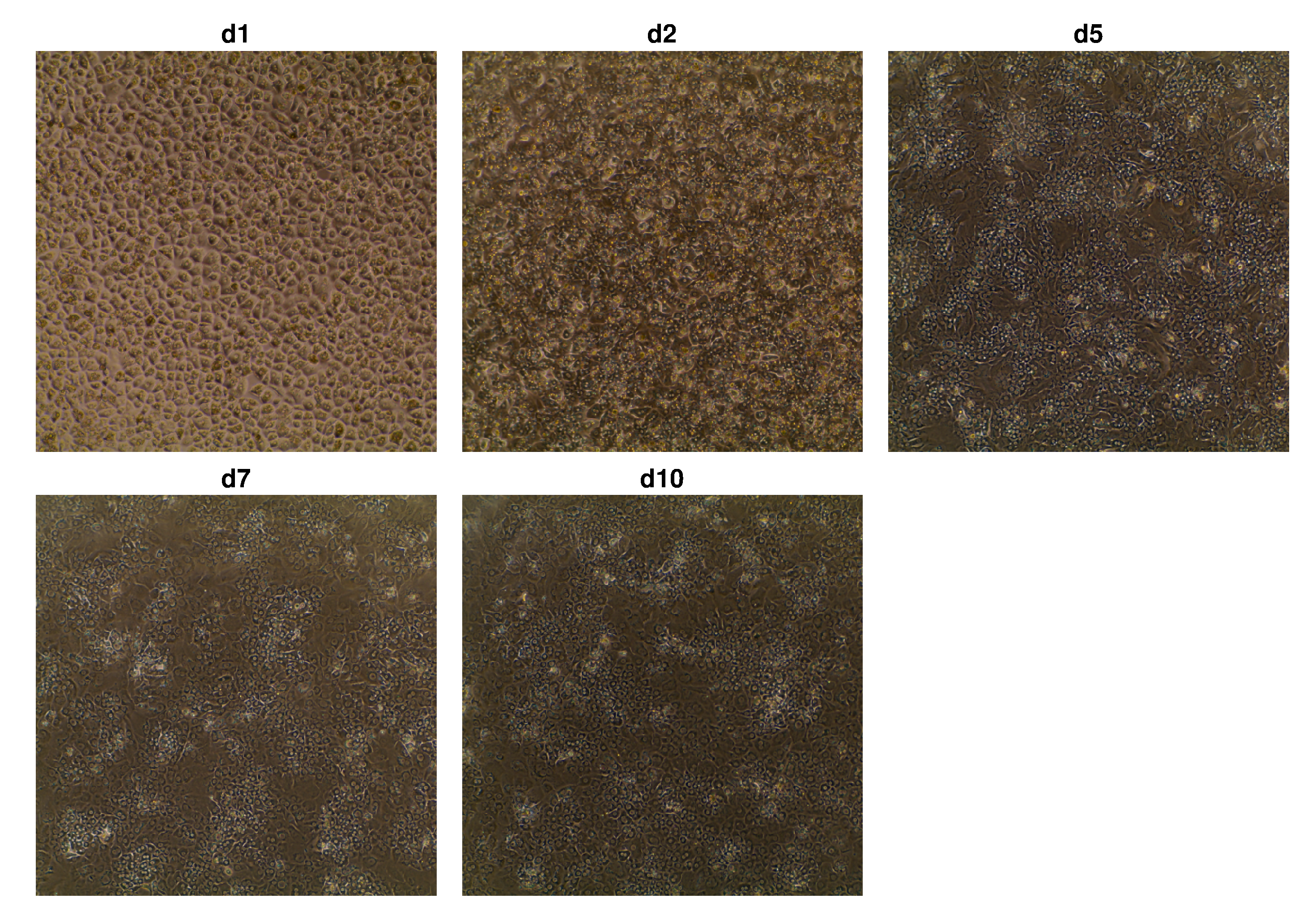

Supplement: S1 Fig — PHH were isolated from liver tissue and seeded into collagen-I-coated culture plates using DMEM/Ham’s F-12 supplemented with 10% FBS, 100U/ml penicillin, 0.1mg/ml streptomycin and 2mM L-glutamine. The medium was changed one day after seeding and then every second day. After two days of culture the medium was supplemented with DMSO (2%) and epidermal growth factor (25ng/ml). Cell morphology was exemplarily visualized by phase contrast microscopy using an EVOSTM XL Core Imaging System (AMG). (TIF) [file pone.0138655.s001.tif]

A

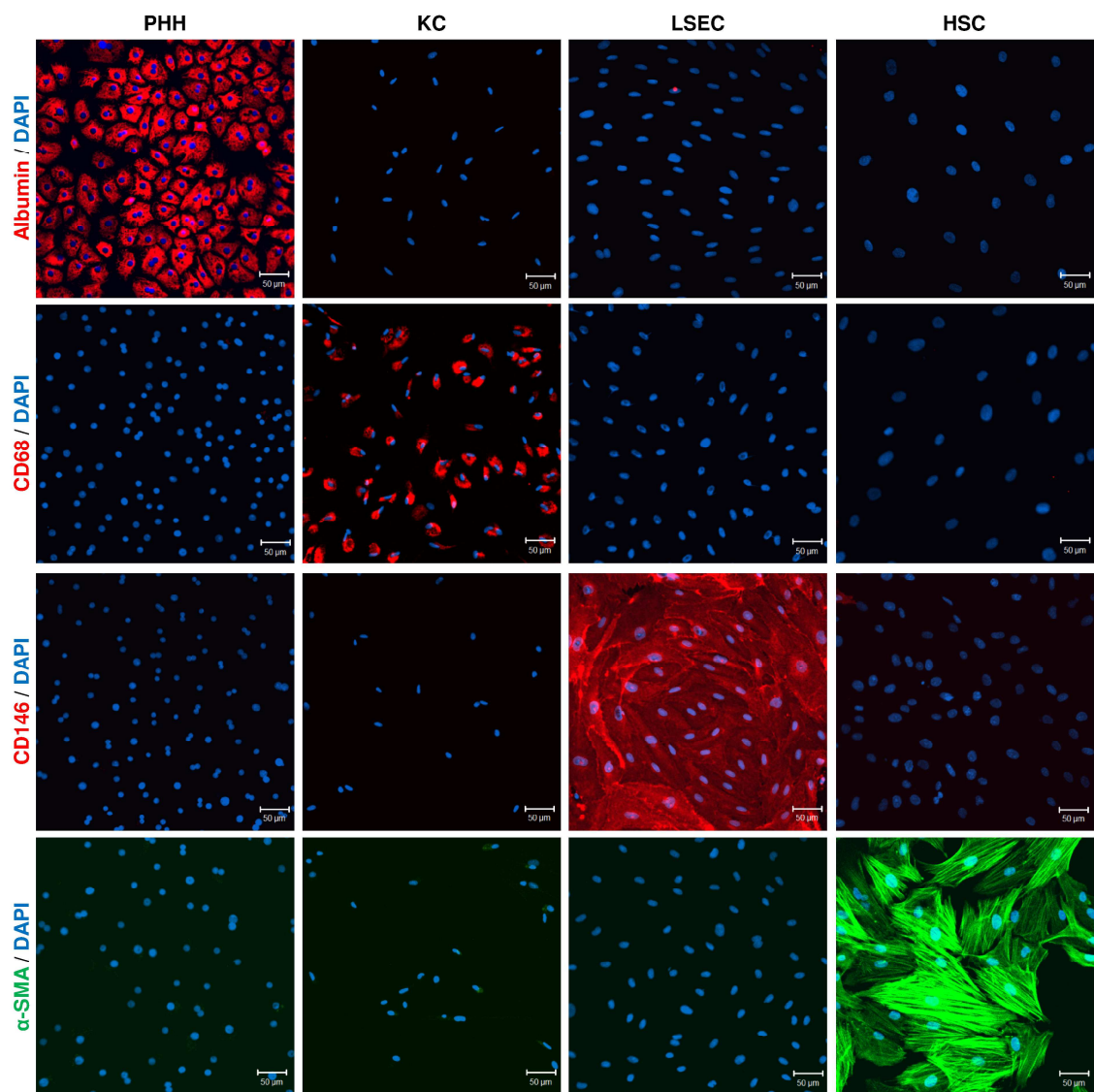

B

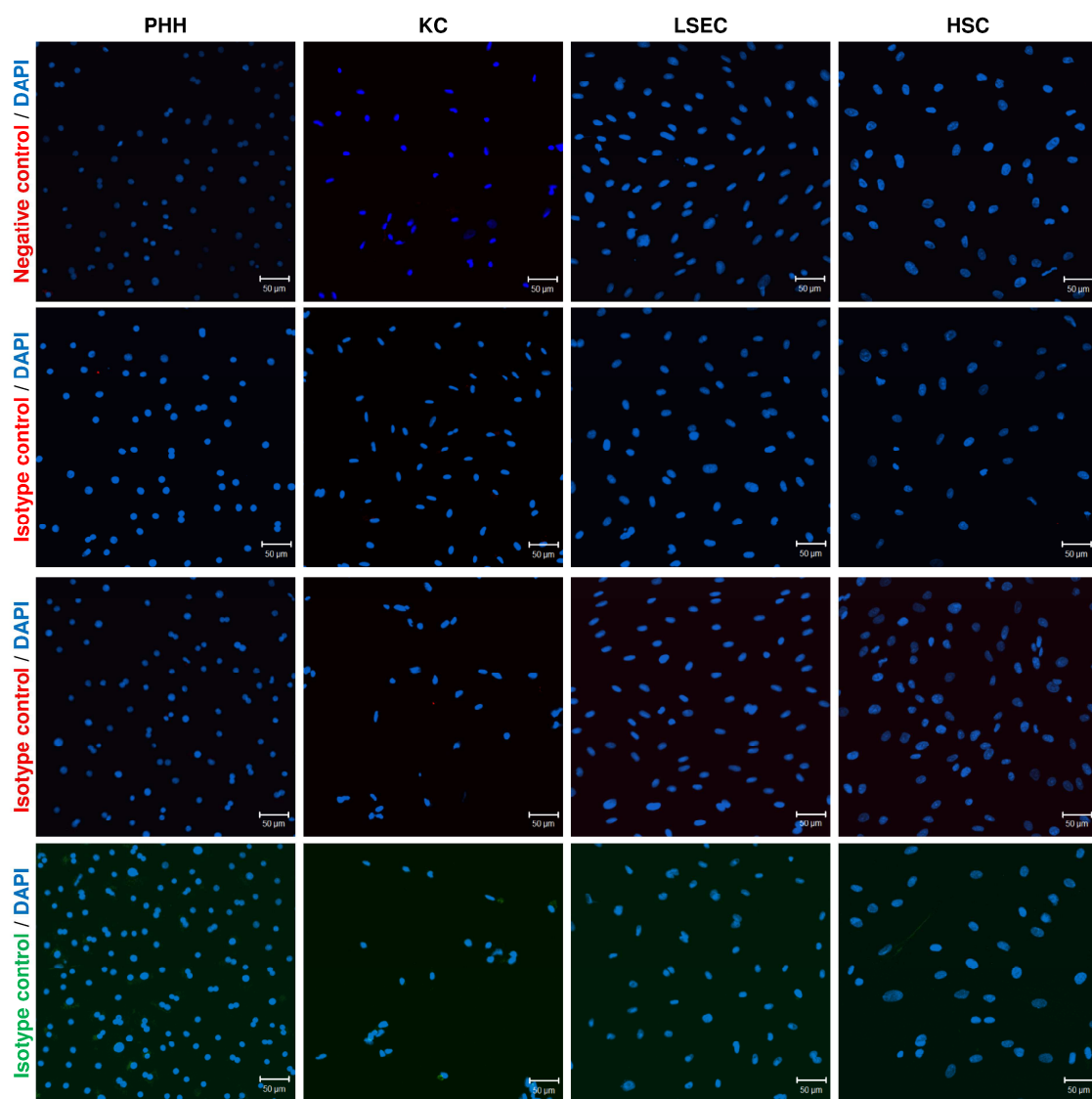

Supplement: S2 Fig — Primary hepatocytes, KC, LSEC and HSC were isolated from human liver tissue (n = 3). Cultured cell populations were immunofluorescently cross-stained for cell type—specific markers (A). Negative controls were performed by omitting the primary antibody (albumin) or using isotype controls (B). Nuclei were counterstained with DAPI (blue). Images were captured at 20× magnification using laser scanning microscope (LSM; Axiovert 100M; Zeiss, Jena, Germany). Scale bar, 50μm. (PDF) [file pone.0138655.s002.pdf]

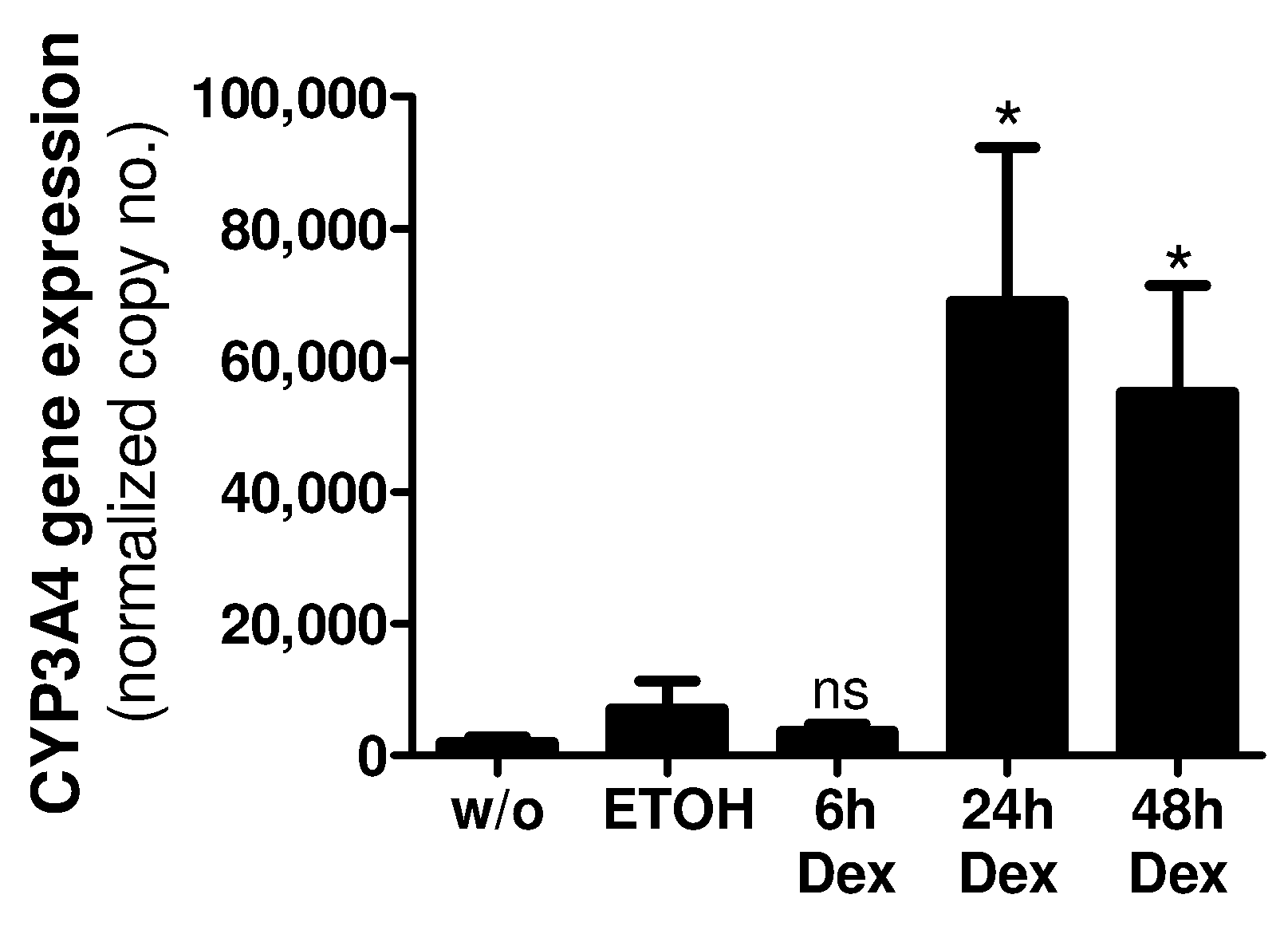

Supplement: S3 Fig — Primary hepatocytes were isolated from human liver tissue (n = 3). One day post preparation PHH were stimulated with 25μM dexamethasone for 6-48h or ETOH for 48h (negative control). RNA was extracted and CYP3A4 gene expression was determined by RT-qPCR. Data represent mean of copy numbers (mean±SEM) normalized to the reference gene ACTB. Asterisks indicate significant results (* p<0.05; ** p<0.01; *** p<0.001). (TIF) [file pone.0138655.s003.tif]

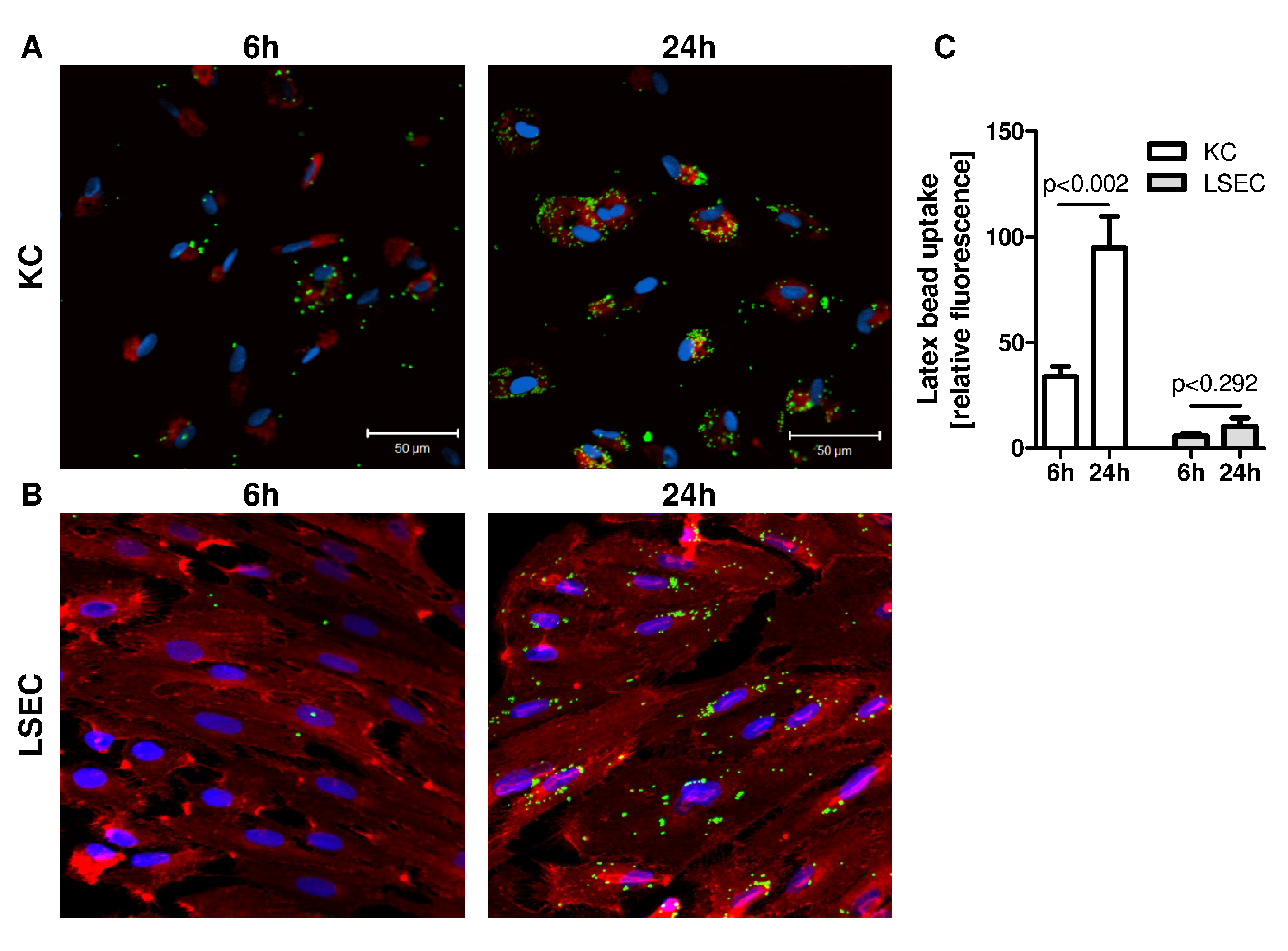

Supplement: S4 Fig — KC (A) and LSEC (B) were incubated with fluorescently labeled latex beads (1μm in size, green) for 6-24h. After incubation cells were fixed and stained for CD68 (KC marker, red) or CD146 (LSEC marker, red), respectively. Nuclei were stained with DAPI (blue). Images were taken at 40× magnification. For quantification of the uptake efficiency intensities of latex bead fluorescence was measured in at least 5 images per cell population (C). (TIF) [file pone.0138655.s004.tif]

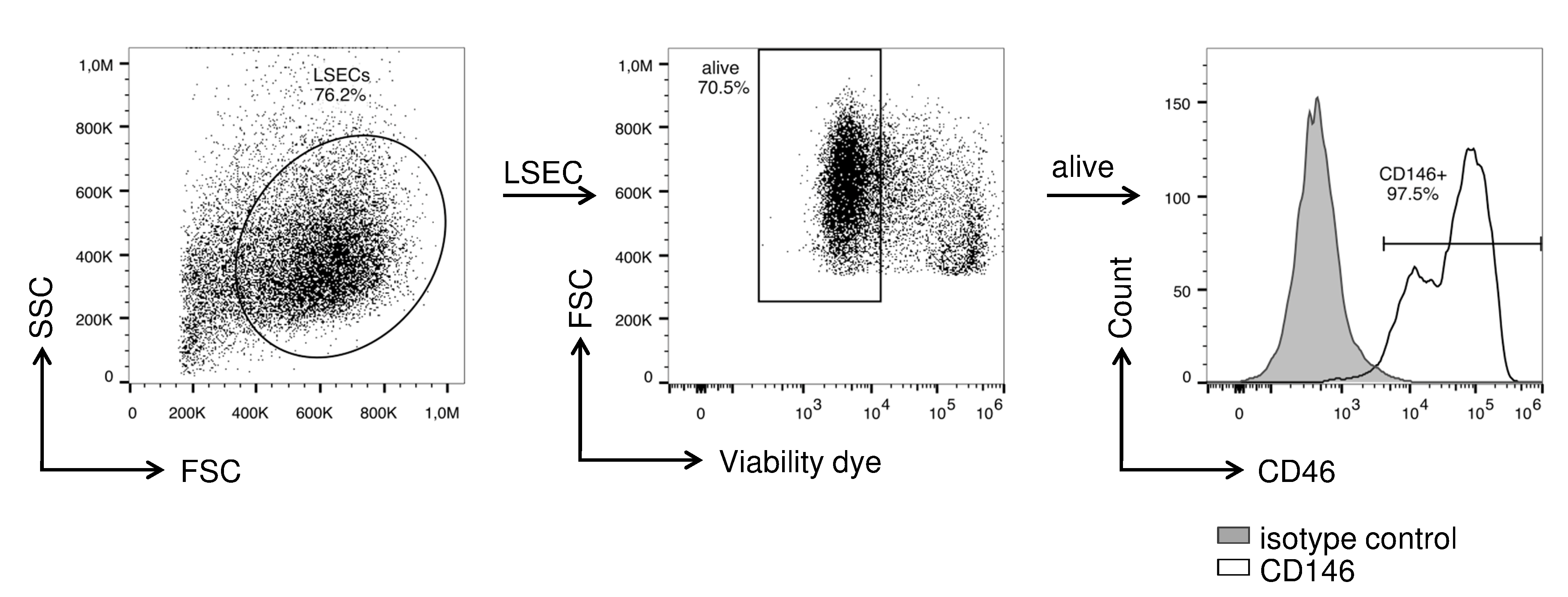

Supplement: S5 Fig — LSEC were stained for CD146 expression and dead cells were excluded by the labelling with a viability dye. Flow cytometry was performed using the Navios flow cytometer (Beckman Coulter, Krefeld, Germany). Result analysis was performed using FlowJo (Treestar, Ashland, Oregon). (TIF) [file pone.0138655.s005.tif]

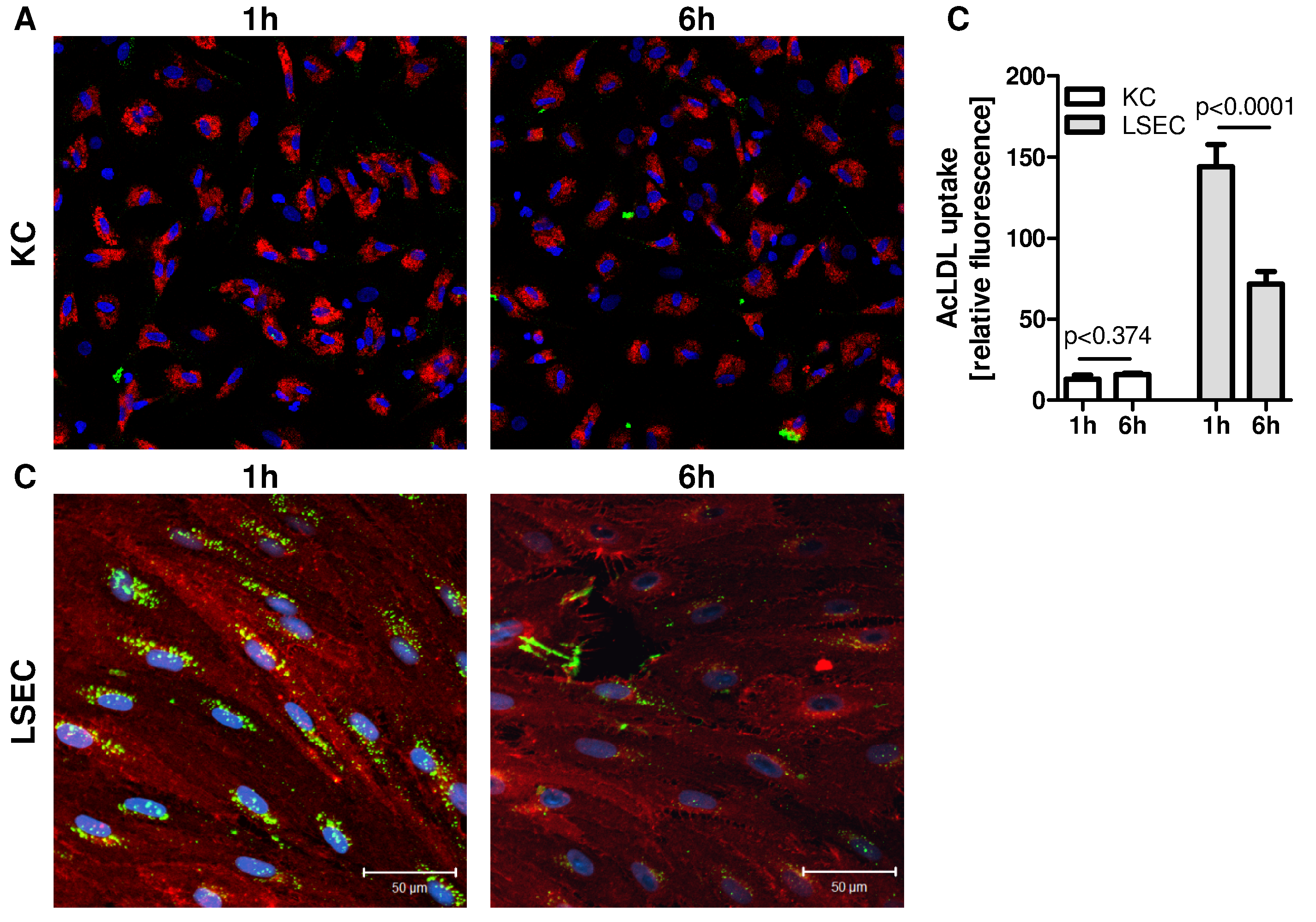

Supplement: S6 Fig — KC (A) and LSEC (B) were incubated with fluorescently labeled AcLDL (green) for 1h and 6h. After incubation cells were fixed and stained for CD68 (KC marker, red) or CD146 (LSEC marker, red), respectively. Nuclei were stained with DAPI (blue). Images were taken at 40× magnification. For quantification of the uptake efficiency intensities of AcLDL fluorescence was measured in at least 5 images per cell population (C). (TIF) [file pone.0138655.s006.tif]
